# Comparative assessment of mouse models for experimental orthodontic tooth movement

**DOI:** 10.1038/s41598-020-69030-x

**Published:** 2020-07-22

**Authors:** Christian Kirschneck, Maria Bauer, Joshua Gubernator, Peter Proff, Agnes Schröder

**Affiliations:** Department of Orthodontics, University Medical Centre of Regensburg, 93053 Regensburg, Germany

**Keywords:** Orthodontics, Molecular medicine, Osteoimmunology

## Abstract

Animal experiments are essential for the elucidation of biological-cellular mechanisms in the context of orthodontic tooth movement (OTM). So far, however, no studies comparatively assess available mouse models regarding their suitability. OTM of first upper molars was induced in C57BL/6 mice either via an elastic band or a NiTi coil spring for three, seven or 12 days. We assessed appliance survival rate, OTM and periodontal bone loss (µCT), root resorptions, osteoclastogenesis (TRAP^+^ area) and local expression of OTM-related genes (RT-qPCR). Seven days after the elastic bands were inserted, 87% were still in situ, but only 27% after 12 days. Survival rate for the NiTi coil springs was 100% throughout, but 8.9% of the animals did not survive. Both methods induced significant OTM, which was highest after 12 (NiTi spring) and 7 days (band), with a corresponding increase in local gene expression of OTM-related genes and osteoclastogenesis. Periodontal bone loss and root resorptions were not induced at a relevant extent by neither of the two procedures within the experimental periods. To induce reliable OTM in mice beyond 7 days, a NiTi coil spring is the method of choice. The elastic band method is recommended only for short-term yes/no-questions regarding OTM.

## Introduction

Induction of orthodontic tooth movement (OTM) by applying mechanical forces to teeth with orthodontic intra- and extraoral appliances requires a coordinated tissue adaptation in the surrounding alveolar bone and periodontal ligament (PDL), a connective tissue, which connects teeth to their bony sockets^1^. Cells present in the bone and PDL, such as osteoblasts, osteoclasts and osteocytes, as well as fibroblasts and cells of the immune system such as macrophages and T cells^2,3^ respond to mechanical forces and changes in the local environment^1^. Their response affects the mass and morphology of the surrounding bone ^4,5^ and triggers a sterile inflammatory reaction at the cellular level leading to increased bone remodelling and turnover^1,6^. In regions of compression within the PDL and the adjacent bone surface osteoclasts are activated, which resorb bone, whereas in regions of tension osteoblasts are activated, forming new bone, thus enabling a therapeutic movement of teeth to correct existing malocclusions^7^.

The interaction of the RANK receptor, its ligand (RANKL) and osteoprotegerin (OPG) plays an important role during OTM^8^. Binding of RANKL, which is expressed by osteoblasts and periodontal ligament fibroblasts, to the RANK receptor, which is found on osteoclast progenitor cells, results in the differentiation of these cells into mature bone-resorbing osteoclasts. OPG competes with the RANK receptor for binding to RANKL and thus inhibits osteoclast differentiation, prevents activation of matrix osteoclasts, and induces their apoptosis^8^. Therefore, bone remodelling is controlled by a balance between RANK-RANKL binding and OPG expression. During orthodontic treatment, increased RANKL levels or an increase in the RANKL/OPG ratio in the sulcus fluid are observed^8–10^. RANKL is mostly secreted by osteoblasts, but during inflammatory processes also periodontal ligament fibroblasts and T lymphocytes contribute to an increased RANKL release, as is the case during OTM or periodontitis^11^.

Since OTM is a multicellular process, it is important to elucidate the biological-cellular mechanisms as well as genetic, endogenous or exogenous influences during OTM or new therapeutic approaches^12^. Due to the numerous cell–cell interactions observed in vivo, animal experiments are often necessary, as isolated examinations of cells ex vivo do not allow clear statements that would be of clinical relevance to the patient. Mammals are chosen because only they allow reasonably comparable results to human metabolism. In basic medical and biological research and literature, experimental orthodontic tooth movement is mostly carried out on rodents^13–16^, which are an established animal model for studies on OTM.

The mouse model has the advantage over the rat model that there are a large number of genetically modified mice available, which makes it possible to investigate genetic influences on OTM. To induce tooth movement in mice mainly two different models are currently reported and regularly used. The first method uses a nickel-titanium coil spring (NiTi spring) inserted between the first upper molar and the upper incisors^15,17,18^, thus moving the first molar into an anterior direction by coil spring relaxation with a constant force level due to the material properties of NiTi (pseudoelasticity). The second method uses an elastic band placed in the interdental space between the first and second upper molar as described by Waldo and Rothblatt^13^. For this purpose, an elastic band is inserted apical of the proximal contact between the first and second maxillary molars. Due to the renewed expansion of the thus compressed elastic band, there is a considerable force acting on the adjacent teeth, which causes them to diverge in a reciprocal manner and thus leads to an experimental tooth movement of the first upper molar in anterior direction.

So far, however, there are no studies that examined these two methods in comparison regarding their suitability and validity for inducing experimental orthodontic tooth movement as well as appropriate time intervals. For this reason, this study deals with the establishment, validation and comparative assessment of these two regularly used methods and associated time intervals for inducing OTM in mice.

## Results

### Animal welfare and survival rate of the orthodontic appliance

**Figure 1:**
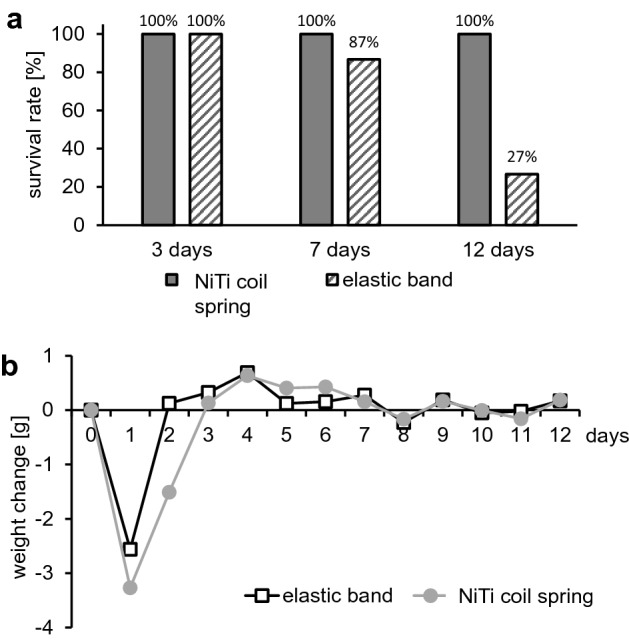
(**a**) Survival rate of the respective orthodontic appliance at the end of each treatment period (3, 7 and 12 days). (**b**) Daily weight change of the animals across the experimental period.

At the respective time points (3, 7 and 12 days) we first checked, whether the corresponding orthodontic appliance (NiTi coil spring vs. elastic band) was still in situ. Three days after insertion of the elastic bands 100% were still in situ, but after 7 days only 86.7% and after 12 days only 26.7% (Fig. 1a). Survival rate of the NiTi coil spring, on the other hand, was 100% for the surviving animals for all time intervals (Fig. 1a), however, 8.9% of the NiTi group did not survive after appliance insertion, respectively. All animals with an elastic band completed the experiment without side effects.

The main indicator of animal welfare was gross body weight assessed daily (Fig. 1b). All animals lost weight after appliance insertion. The extent of this weight loss, however, depended on the intervention. Mice with a NiTi coil spring lost on average 3.6 g of weight (SD = 0.72 g) and those with an elastic band 2.6 g (SD = 0.96 g; Fig. 1b) within the first day. It was noticeable that animals with elastic bands recovered more quickly and regained their initial weight as early as 2 days after the insertion, whereas this was the case only after 3 days for animals with NiTi coil springs (Fig. 1b). After the animals regained their initial weight, slight daily weight fluctuations were observed.

### Relative gene expression of genes involved in inflammation and bone remodelling

**Figure 2:**
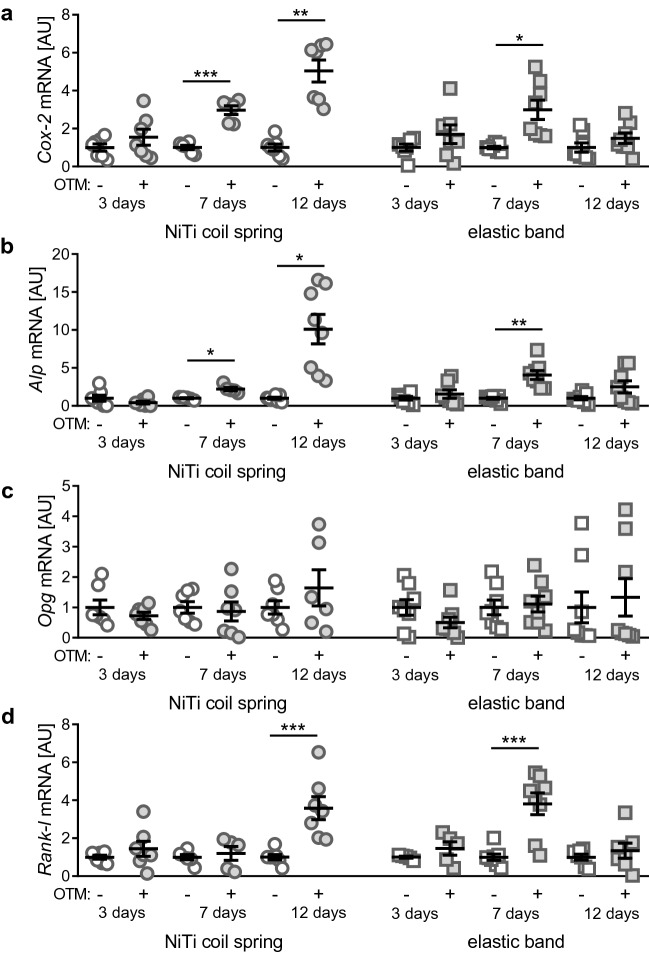
Relative *Cox-2* (**a**), *Alp* (**b**), *Opg* (**c**) and *Rank-l* (**d**) gene expression after three, seven or 12 days of OTM with a NiTi coil spring or an elastic band compared to the untreated contralateral jaw side.[AU] arbitrary units. *p ≤ 0.05, **p ≤ 0.01, ***p ≤ 0.001.

OTM with a NiTi coil spring (Fig. 2a, W = 18.06, DF = 15.52, *p* < 0.0001) produced a statistically significant increase in relative *cyclooxygenase-2* (*Cox-2*) gene expression after both seven (*p* = 0.0009) and 12 (*p* = 0.0023), but not 3 days (*p* = 0.8338) compared to the not orthodontically treated contralateral jaw side. OTM with the elastic band (Fig. 2a, W = 3.361, DF = 16.65, *p* = 0.0277) showed a significant OTM-induced increase in relative *Cox-2* expression after 7 days (*p* = 0.0435), but not after three (*p* = 0.7573) or 12 days (*p* = 0.7598).

OTM with a NiTi coil spring (Fig. 2b, W = 10.39, DF = 14.94, *p* = 0.0002) significantly increased relative *alkaline phosphatase* (*Alp*) gene expression only after seven (*p* = 0.0267) and 12 days (*p* = 0.0176), but not after 3 days (*p* = 0.7687) of application time compared to the not orthodontically treated contralateral jaw side. In contrast, treatment with an elastic band (Fig. 2b, W = 5.284, DF = 17.41, *p* = 0.0039) resulted in enhanced relative *Alp* gene expression only after seven (*p* = 0.0095), but not three (*p* = 0.8790) or 12 days (*p* = 0.5414).

OTM with a NiTi coil spring (W = 0.7461, DF = 15.60, *p* = 0.6009) or an elastic band (W = 1.125, DF = 19.23, *p* = 0.3803) did not induce upregulation of relative *osteoprotegerin* (*Opg*) gene expression after the investigated time periods (Fig. 2c).

OTM with a NiTi coil spring (Fig. 2d, F = 8.860, DF = 33, *p* < 0.0001) significantly increased relative *receptor activator of NF-k ligand* (*Rank-1*) gene expression only after 12 days (*p* < 0.0001), but not after three (*p* = 0.9335) or seven (*p* = 0.9943) days of application time compared to the not orthodontically treated contralateral jaw side. In contrast, treatment with an elastic band (Fig. 2d, F = 10.52, DF = 34, *p* < 0.0001) resulted in enhanced relative *Rank-1* gene expression only after seven (*p* < 0.0001), but not after three (*p* = 0.9708) or 12 days (*p* = 0.9708).

### Effects of both methods for experimental OTM on osteoclastogenesis

**Figure 3:**
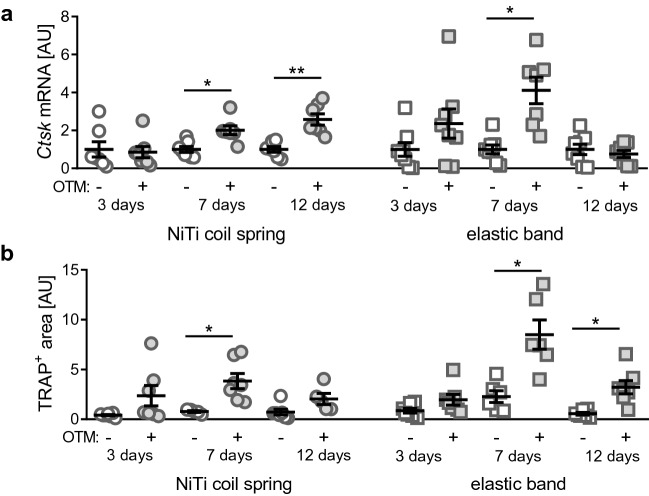
Relative *Ctsk* gene expression (**a**) and relative TRAP^+^ area corresponding to osteoclastogenesis (**b**) after three, seven or 12 days of OTM with a NiTi coil spring or an elastic band compared to the untreated contralateral jaw side. [AU] arbitrary units. *p ≤ 0.05, **p ≤ 0.01.

OTM with a NiTi coil spring (Fig. 3a, W = 6.629, DF = 16.48, *p* = 0.0015) produced a significant increase in relative *cathepsin K* (*Ctsk*) gene expression after both seven (*p* = 0.0376) and 12 (*p* = 0.0098), but not 3 days (*p* = 0.9997) compared to the not orthodontically treated contralateral jaw side with OTM-induced *Ctsk* expression significantly increasing from three to 7 days (*p* = 0.0147), but not from seven to 12 days (*p* = 0.6684). OTM with the elastic band (Fig. 3a, W = 4.134, DF = 18.43, *p* = 0.0090) showed a significant OTM-induced increase in relative *Ctsk* expression after 7 days (*p* = 0.0275), but not after three (*p* = 0.6141) or 12 days (*p* = 0.9736).

OTM with a NiTi coil spring (Fig. 3b, W = 6.678, DF = 13.4, *p* = 0.0025) produced a significant increase in relative TRAP^+^ area and thus osteoclastogenesis after seven (*p* = 0.0486), but not 12 (*p* = 0.3850) or 3 days (*p* = 0.4749) compared to the not orthodontically treated contralateral jaw side. OTM with the elastic band (Fig. 3b, W = 9.290, DF = 14.23, *p* = 0.0004) showed a significant OTM-induced increase in relative TRAP^+^ area after 7 days (*p* = 0.0466) and 12 days (*p* = 0.0477), but not after three (*p* = 0.4863).

### Effects of both methods for experimental OTM on bone morphogenetic factors

µCT evaluation showed a continuous, but insignificant decrease of the interradicular bone density (BV/TV) of the first upper left molar on the treated (OTM) as well as on the untreated side of the jaw over time (Table 1). Insertion of a NiTi coil spring showed no significant effect on BV/TV (F = 5.28, DF = 34, *p* = 0.001), trabecular thickness (TbTh, W = 5.7, DF = 15, *p* = 0.0040), trabecular number (TbN, F = 3.93, DF = 34, *p* = 0.0064) or trabecular spacing (TbSp, W = 3.0, DF = 16, *p* = 0.0502) at any investigated time point (Table 1). By contrast, insertion of an elastic band caused a significant decrease in BV/TV (*p* = 0.0065) and TbN (*p* = 0.0185) only after 7 days while TbTh (*p* = 0.1002) and TbSP (*p* = 0.3993) were not changed significantly (Table 1).

**Table 1:** Bone parameters after treatment with a NiTi coil spring or an elastic band for three, seven or 12 days.

	3 days		7 days		12 day	
	Control	OTM	Control	OTM	Control	OTM
**NiTi coil spring**						
BV/TV [%]	79.0 ± 9.89	69.6 ± 9.77	64.2 ± 10.7	56.3 ± 7.15	54.9 ± 7.15	48.6 ± 19.6
TbTh [mm]	0.26 ± 0.08	0.18 ± 0.06	0.14 ± 0.06	0.12 ± 0.03	0.10 ± 0.02	0.11 ± 0.07
TbN	3.34 ± 0.90	4.03 ± 0.97	4.77 ± 1.01	4.95 ± 0.42	5.58 ± 0.65	5.44 ± 1.92
TbSp [mm]	0.06 ± 0.01	0.08 ± 0.03	0.07 ± 0.02	0.09 ± 0.02	0.08 ± 0.02	0.10 ± 0.03
**Elastic band**						
BV/TV [%]	66.2 ± 13.4	68.5 ± 10.1	68.2 ± 10.7	48.1 ± 11.1**	47.7 ± 12.2	46.7 ± 3.97
TbTh [mm]	0.17 ± 0.09	0.17 ± 0.07	0.16 ± 0.06	0.08 ± 0.02	0.10 ± 0.02	0.09 ± 0.01
TbN	4.37 ± 1.13	4.36 ± 0.84	4.46 ± 0.82	5.73 ± 0.45*	4.94 ± 0.28	5.39 ± 0.52
TbSp [mm]	0.08 ± 0.02	0.07 ± 0.01	0.07 ± 0.02	0.09 ± 0.02	0.11 ± 0.03	0.10 ± 0.01

*BV/TV* bone volume/total volume, *TbTh* trabecular thickness, *TbN* number of trabeculae, *TbSp* trabecular distance.

*p ≤ 0.05, **p ≤ 0.001.

### Effects of both methods for experimental OTM on periodontal bone loss, root resorptions and tooth movement

**Figure 4:**
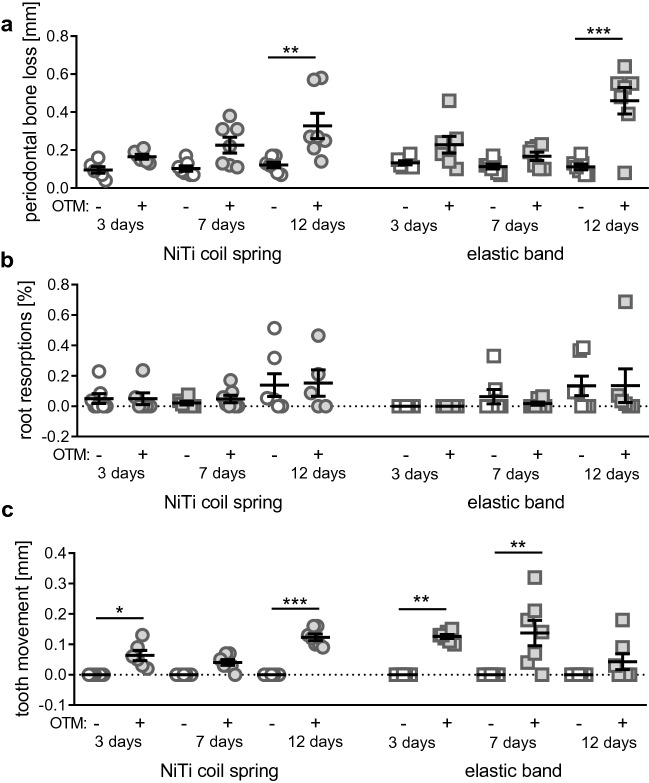
Periodontal bone loss (**a**), orthodontically induced inflammatory root resorptions (**b**) and extent of experimentally induced orthodontic tooth movement (**c**) after three, seven or 12 days of OTM with a NiTi coil spring or an elastic band compared to the untreated contralateral jaw side, at which tooth movement was always 0 mm. *p ≤ 0.05, **p ≤ 0.01, ***p ≤ 0.001.

OTM with a NiTi coil spring (Fig. 4a, F = 6.537, DF = 34, *p* = 0.0002) produced a significant increase in periodontal bone loss after 12 days (*p* = 0.0017), but not three (*p* = 0.6630) or 7 days (*p* = 0.1148) compared to the not orthodontically treated contralateral jaw side. OTM with the elastic band (Fig. 4a, F = 13.84, DF = 36, *p* < 0.0001) showed a significant OTM-associated increase in periodontal bone loss only after 12 days (*p* < 0.0001), but not after three (*p* = 0.3439) or 7 days (*p* = 0.7497).

OTM with a NiTi coil spring (W = 0.9500, DF = 13.61, *p* = 0.4806) or an elastic band (W = 1.679, DF = 14.08, *p* = 0.2040) did not have a significant impact on the extent of orthodontically induced inflammatory root resorptions (OIIRR) after the investigated time periods (Fig. 4b).

The examination of the extent of tooth movement always showed 0 mm on the control side in all examined animals. The NiTi coil spring (Fig. 4c) induced a significant anterior movement of the first upper left molar by on average of 0.06 mm (SD = 0.04 mm) after as early as 3 days (*p* = 0.0382) and a tooth movement of 0.04 mm (SD = 0.03 mm) after 7 days (*p* = 0.1580). After 12 days of OTM with a NiTi coil spring, tooth movement was maximal reaching on average 0.12 mm (SD = 0.03 mm, *p* = 0.0001) As with the NiTi coil spring, the elastic band (Fig. 4c) achieved a significant tooth movement of 0.13 mm (SD = 0.02 mm) after 3 days (*p* = 0.0027). After 7 days, tooth movement with the elastic band was maximal and averaged 0.14 mm (SD = 0.11 mm; *p* = 0.0068). After 12 days of treatment with an elastic band an insignificant tooth movement of on average 0.04 mm (SD = 0.07 mm) could be observed (*p* > 0.9999).

## Discussion

Both tested and validated procedures proved to be suitable to induce experimental orthodontic tooth movement (OTM) in mice. In contrast to the method using a NiTi coil spring, which allowed OTM for up to 12 days with a survival rate of the appliance of 100%, the method employing an elastic band could only be used to induce OTM for up to 7 days, as elastic bands were mostly lost after this time. The insertion of a NiTi coil spring on the other hand was more time-consuming and technically demanding leading a higher risk of injury during insertion associated with a higher loss of body weight after the procedure and a higher mortality rate of animals, whereas all animals receiving the elastic band survived until the end of the experiment. Our data thus indicate that mice recover faster from the insertion of an elastic band than from the insertion of a NiTi coil spring.

Both tested procedures resulted in significant OTM. With the NiTi coil spring, however, a more constant tooth movement than with the elastic band could be achieved with a maximum reached after 12 days, probably still further increasing with longer experimental periods, which were not tested in this study. Our results show that variability of the OTM induced is much higher with the elastic band method than with the NiTi coil spring method, which is probably related to the increased loss of the elastic bands within the experimental periods of seven and 12 days. This method is used in the literature for a maximum of 7 days^19–21^, which coincides with our results, which showed maximum tooth movement within this period, as elastic bands were mostly lost during longer experimental periods. Teeth in an unstable position have an increased risk to return to their starting position^22^. This relapse may have resulted in a backward movement after the loss of the elastic band and reduced the recorded anterior movement of the first upper molar after 7 days explaining the high variability of tooth movement observed. In general two different types of tooth movement can be distinguished—bodily movement, in which the tooth as a whole is changed in its position and tooth tipping^23^. Undesirable or inefficient tooth movement may be due to individual genetic variations or an incorrect application of force. Since both methods resulted in an anterior tipping of the moved first molar, it cannot be ruled out that the tooth movement that has taken place was primarily a tipping movement and to a lesser degree a bodily tooth movement, which is in line with previous reports^16^. This seems to be the case in particular with the elastic band treatment after 3 days. As tooth movement duration is limited for the elastic band method, so is the extent of tooth movement, as once the compressed elastic band has expanded to its original state, it will fall out and OTM terminates or even reverses due to relapse, as discussed before. For scientific experiments depending on an exact and reproducible extent of tooth movement over longer periods of time, the NiTi coil spring method is thus recommended, whereas the elastic band method is rather suitable for short-term experiments (up to 7 days), which require only the induction, but no particular extent of tooth movement.

Clinical studies and reviews generally show no effect of OTM on the alveolar bone level^24,25^. In some studies, however, a larger distance between the cemento–enamel junction (CEJ) and the alveolar bone could be found at the mesial surface of the first and second molars. This was believed to be a direct consequence of orthodontic treatment, either as a response to induced tooth tilt, extruding forces or increased plaque accumulation^26^. These can also be considered as possible causes for the periodontal bone loss demonstrated during this experiment. Periodontal bone loss was found both with the treatment with the NiTi coil spring and elastic band, which increased in analogy to the duration of the test. The most likely explanation for this is the fact that contrary to the clinical situation in humans, both the NiTi coil spring with its wire loop around the first molar and the elastic band positioned below the proximal contact of the first and second upper molar have a distinct traumatizing potential on gingival and periodontal tissues and may advance plaque accumulation at their surface thus propagating periodontal attachment and bone loss. For experimental purposes, however, the extent of periodontal bone loss with both methods is not severe and should not invalidate possible experimental results.

Regarding orthodontically induced inflammatory root resorptions (OIIRR), a common undesirable side effect of OTM affecting up to 80% of orthodontic patients^27^, both methods do not seem to induce significant resorptions at the moved teeth within the respective experimental periods with a clear tendency towards an increased extent of OIIRR discernible after 12 days in both experimental groups. This is in line with previous reports that the extent of OIIRR increases with the duration of OTM^28^.

Cyclooxygenases (Cox) are enzymes that catalyse the first rate-limiting step in prostaglandin synthesis from arachidonic acid^29^. Prostaglandins have a significant function in causing an inflammatory response. They are increasingly synthesized in inflamed tissues and contribute to the development of the typical inflammation signs of redness, warming, swelling, pain and impaired function^30^. Cox-2 plays an important role in a number of physiological and pathological processes^31^. During orthodontically induced tooth movement a sterile inflammation occurs^32^. There is an increase in the concentration of prostaglandin E2 in the sulcus fluid during OTM, which is also an indicator of the processes in the periodontium and highly expressed in both pressure and tension areas of the periodontal ligament^33^. Prostaglandin E2 acts as an inflammatory mediator and activates osteoclasts to effect bone resorption^34^. Since prostaglandin expression depends on the activity of *Cox-2*^30^, an increased expression of this gene can be expected in the course of orthodontic treatment. Induction of orthodontic tooth movement requires a coordinated tissue adaptation in the surrounding bone and periodontal ligament^5,10^. The RANKL-OPG system plays a key role in this^35^. In regions of compression of the periodontal ligament, osteoclasts are activated, which resorb bone. In tension areas of the periodontal ligament, on the other hand, osteoblasts are activated^4,7^. In our study, we observed increased gene expression of *alkaline phosphatase* (*Alp*) as indicator of osteoblast activity^36^. During OTM interaction of the receptor activator of the nuclear factor κB (Rank), its ligand (Rankl) and osteoprotegerin (Opg) plays an important role. By the binding of RANKL to osteoclast precursor cells, these differentiate into mature osteoclasts. OPG, on the other hand, acts as an antagonist to RANK and inhibits osteoclast differentiation through its binding to RANKL^8^. In the course of OTM, there is an increase in the RANKL level, or an increase in the RANKL/OPG ratio in the sulcus fluid^9,10^. Local delivery of OPG leads to an inhibition of osteoclastogenesis and thereby of tooth movement in the corresponding areas^35^. In addition we also tested for osteoclast-specific gene expression and relative TRAP^+^ positive area. Cathepsin K (*Ctsk*) is expressed by osteoclasts^37,38^ and degrade various bone matrix proteins^39^. Due to bone remodelling taking place during OTM, there was consequently an increase in the expression of *Ctsk* and relative TRAP^+^ positive area^40^.

Both the NiTi coil spring and the elastic band method induced an enhanced relative gene expression of *Alp*, *Rankl* and *Cox-2*. Furthermore, we observed an increased relative *Ctsk* gene expression and TRAP^+^ area with both methods, indicating an increased osteoclastogenesis and osteoclast activity. In line with the extent of OTM observed, a maximum expression was reached for most genes after 12 days for the NiTi coil spring method and after 7 days for the elastic band method. Interestingly, after 3 days of OTM, no significant upregulation of relative gene expression of *Alp*, *Rankl, Cox-2* or *Ctsk* or relative TRAP^+^ area corresponding to osteoclastogenesis could be observed for both methods. This indicates that the tooth movement observed within 3 days was not an actual biological tooth movement based on bone remodelling processes, but was rather achieved by mechanical deformation of the related hard and soft tissues (bone, tooth, periodontal ligament etc.) by the applied force and was thus most likely reversible at this point, if the respective appliance had been removed.

## Conclusion

In order to induce experimental orthodontic tooth movement in the mouse model, both tested procedures (NiTi coil spring and elastic band) are suitable, but both have respective strengths and weaknesses. Advantages of the elastic band method are the easy and fast insertion of the elastic bands resulting in a smaller amount of narcotic required and increased animal welfare and survival, thus representing a lower burden for the animals. Disadvantages are the not exactly determinable force level acting on the teeth, the short duration and limited extent of orthodontic tooth movement for at maximum 7 days compared to at least 12 days for the NiTi coil spring method and the large interindividual variation of the tooth movement achieved. Side effects such as periodontal bone loss and orthodontically induced root resorptions (OIIRR) were not induced at a relevant extent by neither of the two procedures tested within the respective experimental time periods.

## Materials and methods

### Study design and experimental animals

**Figure 5:**
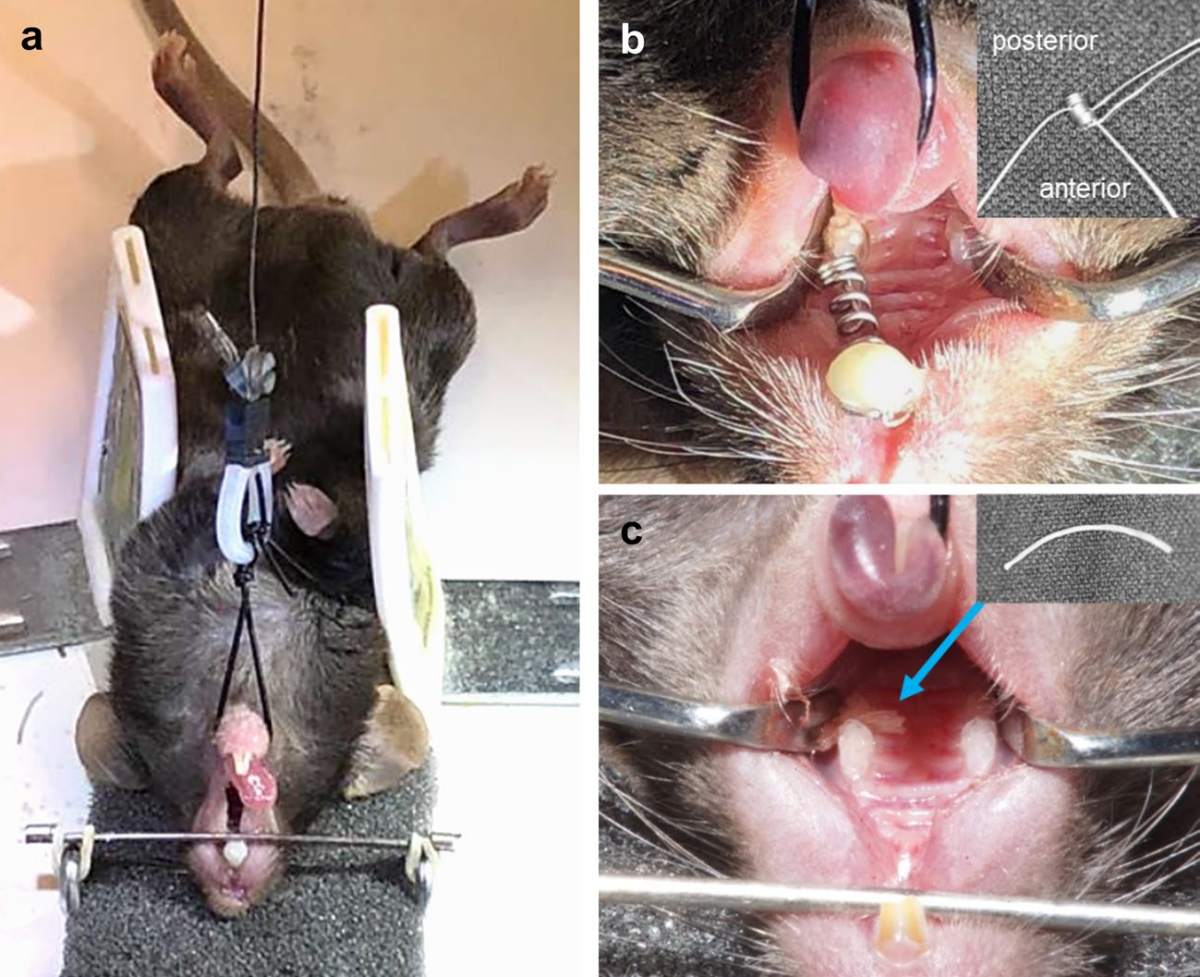
(**a**) Custom-made fixation apparatus for orthodontic interventions in mice. Details see manuscript text. (**b**) Halved 0.25 N NiTi coil spring (*Inset:* auxiliary wires inserted into spring ends) between the first upper left molar and the upper incisors for experimental anterior tooth movement of the first upper left molar. (**c**) Elastic band (Ø 0.3 mm, see arrow, *Inset*) between the first and second upper left molar for experimental anterior tooth movement of the first upper left molar.

A total of 90 male C57BL/6N wild-type mice (age: 8–10 weeks, Charles River, Fig. 5a) were randomly assigned to one of two different orthodontic procedures to induce experimental orthodontic tooth movement (OTM). 45 C57BL/6 N mice received a halved NiTi coil spring (10–000-26, Sentalloy, GAC International, Fig. 5b) and 45 C57BL/6 N mice an elastic band (Inwaria, thread elastic Ø 0.3 mm; Fig. 5c), respectively, for anterior movement of the first upper left molar. The contralateral untreated jaw side served as a non-force internal control (split-mouth model) and all measurements were performed at both jaw sides. 15 mice were included in each group. For micro-CT and histological analyses seven animals (n = 7) per time point and for RT-qPCR analyses eight animals per time point (n = 8) were examined. After 3, 7 or 12 days of OTM, the mice were euthanized according to legal guidelines. Sample sizes were based on a previous study^41^ using a similar experimental setup to induce orthodontic tooth movement in rats by means of a NiTi coil spring as well as a similar technique of retrieving samples and performing RT-qPCR analyses, which proved to provide sufficient study power, while minimizing animal suffering.

The animal experiments were carried out with the approval of the responsible authorities (AZ: 55.2-2532-2-567) and in compliance with the German Animal Protection Act. In order to avoid unnecessary suffering of the animals, corresponding termination criteria were predefined and animal condition as well as gross body weight monitored daily. The animals were kept in a conventional S1 animal laboratory at the University of Regensburg. The laboratory maintains a temperature of 21 °C and a relative humidity of 55% with a 12-h light–dark rhythm with the light phase between 7:00 h and 19:00 h. The experimental animals were kept in groups of six in transparent polycarbonate cages with a metal grid cover. The mice had ad libitum access to distilled water in plastic bottles, which was renewed twice a week, and to a standard diet (V1535, ssniff) with feed pellets softened to mash from the beginning of orthodontic treatment and placed in a feeding trough inside the cage. After an acclimatisation phase of at least one week after delivery, the experiment was started so that the baseline age of the animals was 9–10 weeks with an average weight of 24.4 g (SD 1.2 g).

As anaesthetic, xylazine/ketamine was used in the ratio 1: 3, which was diluted 1: 5 with physiological saline. Animals receiving a feather were injected intraperitoneally with 0.008 ml of the dilution per gram of body weight. The animals treated with an elastic band received standardized 0.1 ml of the dilution, because they had to be anesthetized for a much shorter period of time.

For treatment, animals were positioned on their back in a custom-made apparatus (Fig. 5a), which was modified from a previously used system in rats^16^. The upper jaw was immobilised by an anatomical probe spanning across the palate between the upper incisors and molars, which is connected to the platform of the apparatus by rubber bands, thus pressing the head firmly to the platform. To enable easy access to the oral cavity for interventions, the mandible and tongue were retracted from the immobilised upper jaw until an adequate mouth opening was achieved by means of a wire loop around the mandibular anterior teeth and tongue, fixated via a connecting wire with an inserted coil spring as stressbreaker in the appropriate tensed state at a vertical beam at the back of the platform.

### Experimental tooth movement with a NiTi coil spring

A NiTi coil spring with the lowest commercially available force level of 0.25 N (10-000-26, Sentalloy, GAC International) and a length of 3 mm was cut in half, as due to the limited available space between the upper mouse molars and incisors a tensioning of the spring would otherwise not have been possible. Subsequently, two orthodontic stainless steel auxillary wires (Ø 0.08 mm) were threaded directly into the spring, as described previously^16^ (Fig. 5b, Inset). To ensure a uniform force level of about 0.35 N by spring extension as recommended by Taddei et al.^42^, which was quantified and adjusted upon insertion with a calibrated orthodontic spring balance (Correx, small model, Haag-Streit AG, Köniz, Switzerland) as described before^41^, care was taken to ensure that there are always exactly four coils between the two wires. The spring was attached to the neck of the first left upper molars with a wire loop initially threaded through the proximal space between the first and second upper molars from palatally (Supplementary Fig. 1a) enclosing both the first molar and the end of the spring with its inactive coils (Supplementary Fig. 1b). Then the spring was stretched to the upper incisors and the auxillary wire inserted at the anterior end encompassing the terminal inactive coils was threaded through the approximal space of the maxillary incisors (Supplementary Fig. 1c), passed around the two incisors, then guided through the inactive anterior terminal coils of the spring at the neck of the incisors, and again threaded through the approximal space of the incisors (Supplementary Fig. 1d). There, the two wire ends could be briefly attached to the holding apparatus with an adhesive tape in order to avoid a change in position. The final anterior attachment to the incisors was achieved by means of layer of flowable composite over the wire loop and the anterior teeth. For this purpose, the surface of the incisors was etched with 37% phosphoric acid (i-Bond Etch 20 gel, Heraeus Kulzer) for 120 s, rinsed for 15 s and dried with an air syringe for 15 s. Subsequently, Transbond XT primer (3 M Unitek) was applied by means of a microbrush and light-cured for 20 s. Subsequently, a thin flowable composite coating (Tetric EvoFlow A3, Ivoclar Vivadent) was applied to the wire loop and the tooth, shaped with a dental probe and light-cured for 40 s. A small oral extension of the coating encompassing the terminal inactive end of the coil spring was formed to protect the delicate transition between the composite coating and the NiTi spring during mastication. Afterwards the auxillary wire ends fixated with the adhesive tape could be loosened, twisted around each other (Supplementary Fig. 1e), shortened, bent laterally and covered again with some additional flowable composite to avoid injuries from a sharp wire end (Supplementary Fig. 1f, Fig. 5a). The mandibular anterior teeth were cut at papillary level once upon appliance insertion with a diamond-plated cutting disc at 10,000 rpm to prevent damage to the spring by the lower incisors during mastication.

### Experimental tooth movement with an elastic band

For the investigation of the Waldo/Rothblatt method, an elastic band with a diameter of 0.3 mm (Inwaria) was used (Fig. 5c, Inset), which was the largest diameter insertable in the proximal space for maximal experimental tooth movement. First, the proximal space between the first and second upper molars was pre-expanded with an orthodontic auxillary wire (Ø 0.08 mm), then the elastic band was inserted using two Mosquito clamps (straight, with teeth) and then shortened accordingly at both sides (Fig. 5c).

### µCT analyses for evaluation of periodontal bone properties/loss and tooth movement

Seven animals per experimental group and timepoint were perfused with 5% formaldehyde and upper jaws disected and stored overnight in 5% formaldehyde. Jaws were then transferred to 0.1% formaldehyde for long-time storage until µCT measurements were performed, which were carried out at the OTH Regensburg with the device Phoenix vltomelxs 240/180 (GE Sensing & Inspection Technologies), with a 180 kV-NF tube using the following settings: Voxelsize: 10 μm, Images: 1800, Timing: 333 ms, Voltage: 50 kV, Current: 750 μA, Fastscan: Scan duration 10 min. Image reconstruction and evaluation was performed with the software Volume Graphics—VG Studio Max (Volume Graphics GmbH). These were all performed within a sagittal layer plane, aligned perpendicular to the occlusal plane, parallel to the palatal suture and placed through the middle of the first molar at the level of the cemento–enamel junction (CEJ) in order to allow reproducible measurements. To determine the properties of the alveolar bone, an interradicular cube was created, by using the “regions of interest” (ROI) function. The edge lengths of the inserted cube were 0.35 × 0.35 × 0.35 mm (height × width × depth, Supplemental Fig. 2). These specified values were exported as a file, saved and imported for all other µCT images to be measured and adopted as a measurement variable. As due to minimally varying tooth root morphology of the first upper molar from animal to animal an exact landmark-based approach to place the cubical region of interest was not possible, we set the cubical region of interest manually-visually in the central interradicular region 0.2 mm below the furcation taking special care not to transgress into other non-bony structures such as the tooth roots. After extracting the data, the cube could be viewed and evaluated as a volume. Periodontal bone loss at the first molar was measured distally along the root surface as an increase of the distance between the cemento–enamel junction and the alveolar limbus (Supplemental Fig. 3a). To quantify the extent of orthodontic tooth movement, the smallest distance between the crowns of the first and second upper molar was measured with the vernier caliper function of the software (Supplemental Fig. 3b).

### Preparation of histological samples

After µCT analyses jaws were divided into the control and orthodontically-treated (OTM) side and demineralised in Tris-buffered ethylene diamine tetra-acetic (EDTA) solution (10%, pH = 7.4)^43^ for eight weeks at room temperature. Jaws were then embedded in paraffin and cut in sagittal-oblique sections of the tooth-bearing alveolar process of approximately 5 µm using a rotating microtome (HM350, Microm International). All sections were mounted onto Superfrost glass slides (SuperFrost Plus).

### HE staining for evaluation of orthodontically induced inflammatory root resorptions

Sections were deparaffinised at 60 °C for 30 min and directly transferred to xylene (9713.2, Carl-Roth) for 20 min. Sections were hydrogenated by a descending series of alcohol consisting of twice 100% ethanol, 96% ethanol, 70% ethanol and H_2_O_dd_ for 10 min each. This was followed by staining of the cell nuclei with Mayer haematoxylin solution (1.07961.0500, Sigma-Aldrich) for 10 min. Afterwards, slides were incubated under running, warm water for 5 min. This was followed by counterstaining with freshly prepared eosin G solution 0.5% (X883.2, Carl-Roth) for one minute. Thereafter, the sections were rinsed under warm tap water and dehydrated by the ascending series of alcohol, with dips in the 70% and 96% ethanol sections only briefly to avoid discoloration. After a residence time of at least 20 min in xylene, the coverslips were applied with entellan (1.07961.0500, Merck). The stained histological sections were photographed and digitized under the microscope (Keyence BZ-X800, Neu-Isenburg, Germany, Supplemental Fig. 4). The relative extent of orthodontically induced inflammatory root resorptions (OIIRR) at the mesio-buccal root of the first upper molar was quantified with the software ImageJ (Ver.147, National Institutes of Health, USA) as total resorption area to total root area (between the CEJ and the apex of the furcation) as described before^44^.

### TRAP (tartrate-resistant acid phosphatase) staining for evaluation of osteoclastogenesis

Paraffin sections were incubated overnight at 37 °C and then hydrogenated via a descending alcohol series (compare HE staining) and rinsed in H_2_O_dd_. Slides were placed for 10 min at room temperature in a previously freshly prepared TRAP buffer consisting of 1.64 g sodium acetate (6773.1, Carl-Roth) and 23 g of di-sodium tartrate dihydrate (T110.1, Carl-Roth) to 500 ml of H_2_O_dd_. pH was adjusted to 5 with HCl. Sections were then placed in a freshly prepared staining solution consisting of 40 mg of Naphtol AS-MX Phosphate Disodium Salt (N5000, Sigma-Aldrich), 4 ml of N,N-dimethylformamide (D4551, Sigma-Aldrich), 240 mg of Fast Red Violet LB Salt (F3381, Sigma-Aldrich), 2 ml Triton X-100 (T9284, Sigma-Aldrich) and 200 ml of the previously prepared TRAP buffer. The sections were incubated at 37 °C for two hours, then rinsed in H_2_Odd and counterstained for 3 min with filtered Mayer's haematoxylin solution (51275, Sigma-Aldrich) at room temperature. Subsequently, the sections were covered immediately with Aquatex (1085620050, Merck). Stained histological sections were photographed under the microscope (Keyence BZ-X800, Neu-Isenburg, Germany, Supplemental Fig. 5). The relative extent of the TRAP-positive area corresponding to osteoclastogenesis and osteoclast activity within the periodontal ligament and adjacent bone and tooth surfaces at the mesio-buccal root of the first upper molar was quantified with the software ImageJ (Ver.147, National Institutes of Health, USA) using the "Color Threshold" function (Hue 189-255, Saturation 50 255, Brightness 0-203, Method: Default) as TRAP-positive area to total root area (area between the CEJ and the apex of the furcation) as described before^44^.

### RNA isolation, reverse transcription and RT-qPCR analysis

A cuboid tissue sample of defined extension was prepared of eight animals per experimental group and timepoint with a sterile cutter by macrodissection, containing the first upper left or right molar without the clinical crowns with adjacent periodontal tissue and alveolar bone as described in Kirschneck et al.^41^, immediately transferred into liquid nitrogen and stored at − 80 °C until RNA isolation could be performed using the RNeasy Mini Kit (74104, Qiagen) according to the manufacturer’s instructions. Before RNA extraction, samples were refrozen in liquid nitrogen and then pulverized in a bone mill (Retsch). The recovered RNA was finally quantified and checked for purity as described before^41^ with a nano-photometer (Implen). For cDNA synthesis RNA was added to nuclease free water depending on the concentration. 11 μl of this solution was added to 9 μl of a previously prepared master mix. This consisted of random hexamer primer (0.1 nmol, 1 μl, SO142, Life Technologies), oligo-dT18 primer (0.1 nmol, 1 μl, SO131, Life Technologies), 5 × M-MLV Buffer (4 μl, M1705, Promega), dNTP mixture (40 nmol, 1 μl–10 nmol/dNTP, L785.2, Carl Roth), reverse transcriptase (200 U, 1 μl, M1705, Promega) and RNase inhibitor (40 U, 1 μl, EO0381, Life Technologies). The samples were incubated for 1 h at 37 °C, followed by an incubation at 95 °C for 2 min to inactivate the reverse transcriptase. Subsequently, the cDNA was stored at − 20 °C until further processing. RT-qPCR amplification reaction was carried out as described before^41^ in a Mastercycler ep realplex thermocycler (Eppendorf) with 96-well PCR plates (712282, Biozym Scientific) and adhesive optical seal film (712350, Biozym Scientific). SYBR Green JumpStart Taq ReadyMix (7.5 μl, S4438, Sigma-Aldrich), the respective primer pair (0.75 μl per primer; Table 2) and 1.5 μl of the corresponding cDNA were filled up to a total volume of 15 μl with nuclease-free water (T143, Carl-Roth). C_q_ values were identified as second derivative maximum of the fluorescence signal curve employing the software realplex (version 2.2, EppendorfAG, CalQPlex algorithm, Automatic Baseline, Drift Correction On). Evaluation of the RT-qPCRs was carried out by the method of relative quantification as described before^41,44,45^. Briefly, the expression of the target genes was related to that of the reference gene *Eef1a1* and defined as 2^−ΔCq^ with ΔC_q_ = C_q_ target gene − C_q_ (*Eef1a1*). Prior to statistical analysis of relative gene expression data, all data values were divided by the respective arithmetic mean of the not-orthodontically-treated jaw side at each timepoint for each treatment to obtain normalized data values relative to these controls, set to 1.

**Table 2:** Primer sequences of target genes and reference gene *Eef1a1* used in this study.

Gene symbol	Gene name	Accession number	5′-forward primer-3′	5′-reverse primer-3′
*Eef1a1*	Eukaryotic translation elongation factor 1 alpha 1	NM_010106.2	AAAACATGATTACAGGCACATCCC	GCCCGTTCTTGGAGATACCAG
*Alpl*	Alkaline phosphatase	NM_007431.3	GGGGTACAAGGCTAGATGGC	AGTTCAGTGCGGTTCCAGAC
*Cox2*	Cyclooxygenase 2	NM_011198.4	TCCCTGAAGCCGTACACATC	TCCCCAAAGATAGCATCTGGAC
*Ctsk*	Cathepsin K	NM_007802	GACCCATCTCTGTGTCCATCG	CCATAGCCCACCACCAACAC
*Opg*	Osteoprotegerin	NM_008764.3	AGAAGCCACGCAAAAGTGTG	TTGGTCCCAGGCAAACTGTC
*Rank-l*	Receptor activator of NF-kB ligand	NM_011613.3	CGACTCTGGAGAGTGAAGACAC	ACCATGAGCCTTCCATCATAGC

### Data analysis and statistics

Statistical evaluation was performed with the program GraphPad Prism (Version 8.00 for Windows, GraphPad Software). Normal distribution was tested with a Shapiro–Wilk test. Depending on distribution of data either welch-corrected ANOVAs followed by Games-Howell multiple comparison test or ordinary ANOVAs followed by Holm Sidak’s multiple comparison tests were performed. Extent of tooth movement was analysed using Kruskal–Wallis test followed by Dunn’s multiple comparison test. The graphs show the individual data values as well as the mean ± standard error of the mean. Significance was assumed at *p* ≤ 0.05.

## Supplementary information

Supplementary information.

## Data Availability

All datasets are publically available either as supplementary information to this article or upon request from the corresponding author.
