# Supplementary material for: Comparative assessment of mouse models for experimental orthodontic tooth movement
Source: Sci Rep. 2020 Jul 22;10:12154. doi: 10.1038/s41598-020-69030-x (PMC7376195; doi:10.1038/s41598-020-69030-x)
Supplement: Supplementary file 1 — Supplementary information. [file 41598_2020_69030_MOESM1_ESM.pdf]

Comparative assessment of mouse models for experimental orthodontic tooth movement

Christian Kirschneck*, Maria Bauer, Joshua Gubernator, Peter Proff, Agnes Schröder

Department of Orthodontics, University Medical Centre of Regensburg, Regensburg, D-93053, Germany

*corresponding author, christian.kirschneck@ukr.de, Tel.: +49 941 944 6093; Fac.: +49 941 944 6169

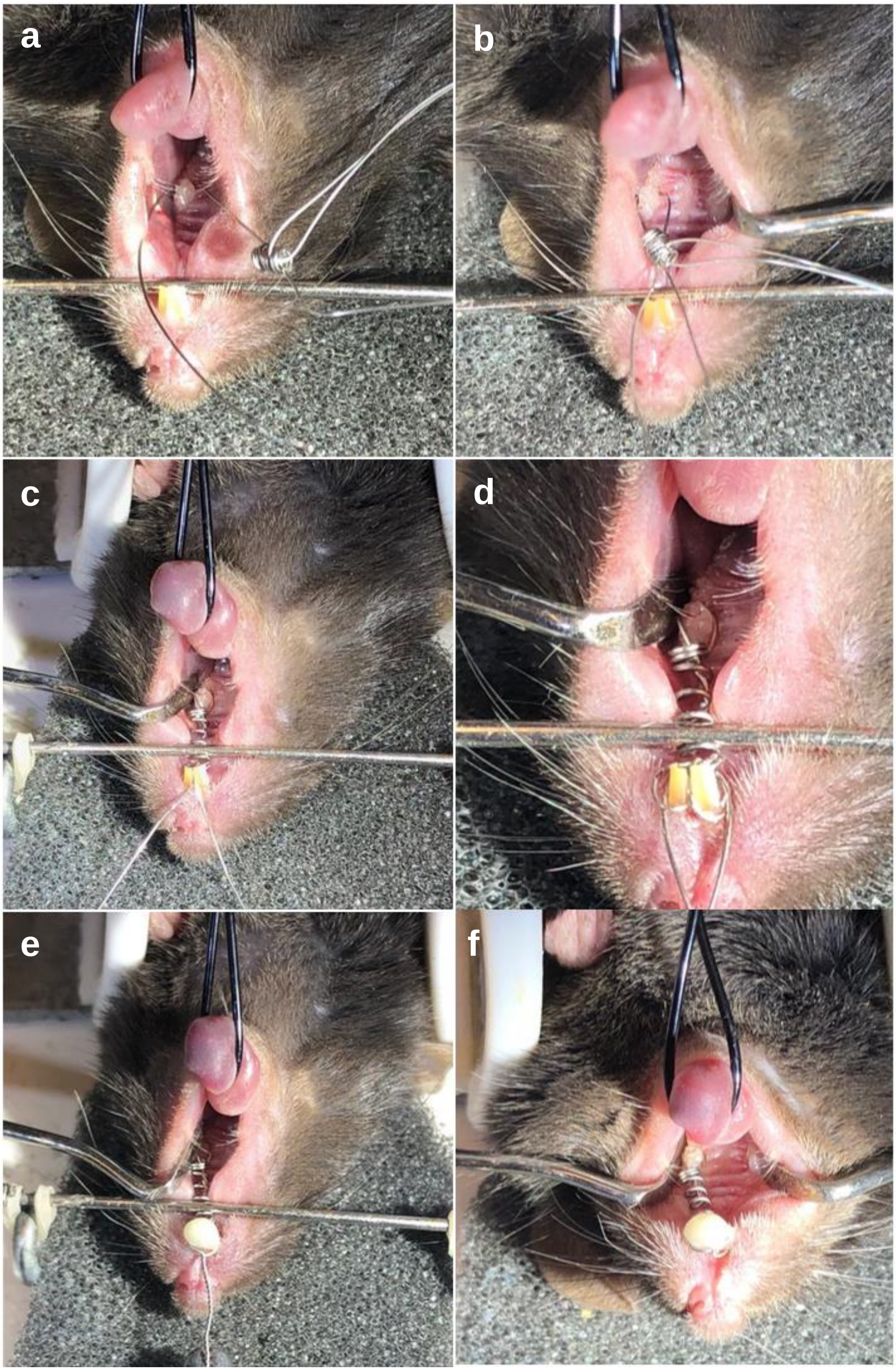

Supplemental Figure 1. Intraoral insertion of the NiTi coil spring. (a) Introduction of the wire ligature between the first and second upper left molars. (b) Threading of the wire through the spring. (c) The wire is guided through the approximal space of the anterior teeth. (d) Fixation of the wire around the anterior teeth. (e) Fixation with flowable composite and subsequent twisting of the wire. (f) NiTi coil spring *in situ*. Details see manuscript text.

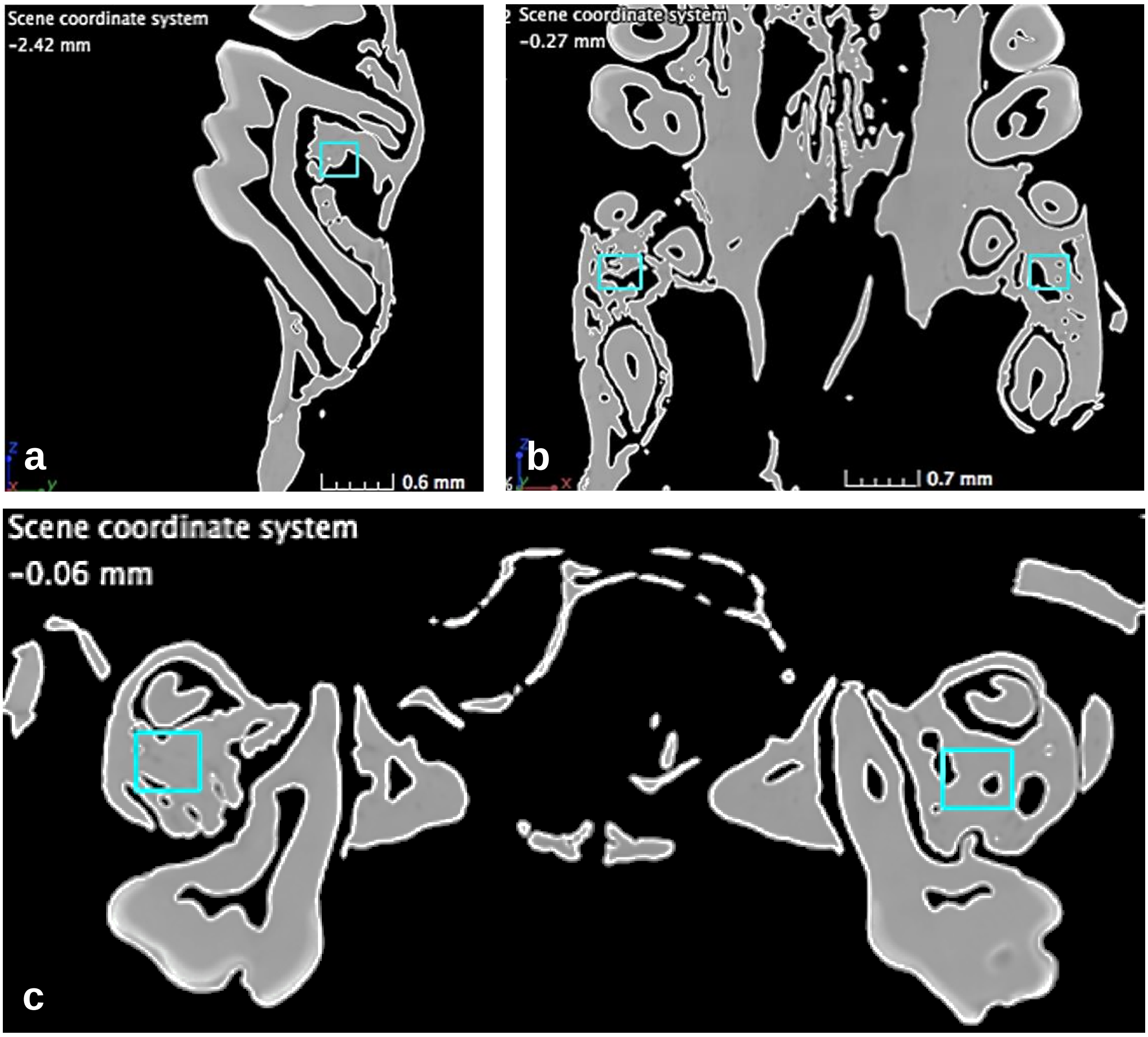

Supplemental Figure 2. Interradicular, cube-shaped (edge length 0.35 mm) "region of interest" (ROI) at the first upper molar within the microCT image for assussment of bone volume / total volume (BV / TV), trabecular thickness (TbTh), number of trabeculae (TbN) and trabecular distance (TbSp). Sagittal (a), axial (b) and coronal (c) view.

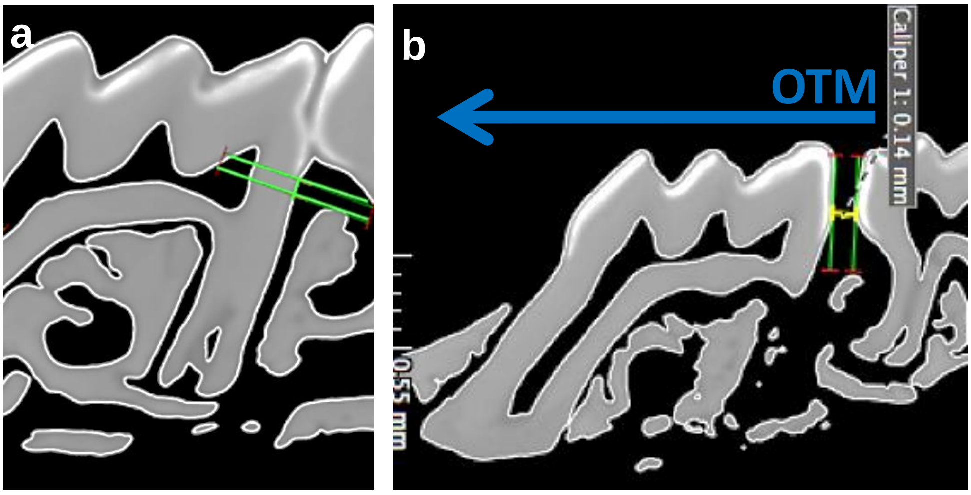

Supplemental Figure 3. (a) Periodontal bone loss at the first upper molar measured distally along the root surface as an increase of the distance between the cemento-enamel junction and the alveolar limbus (short green line). (b) Extent of orthodontic tooth movement as smallest distance (yellow) between the crowns of the first and second upper molar. OTM = orthodontic tooth movement

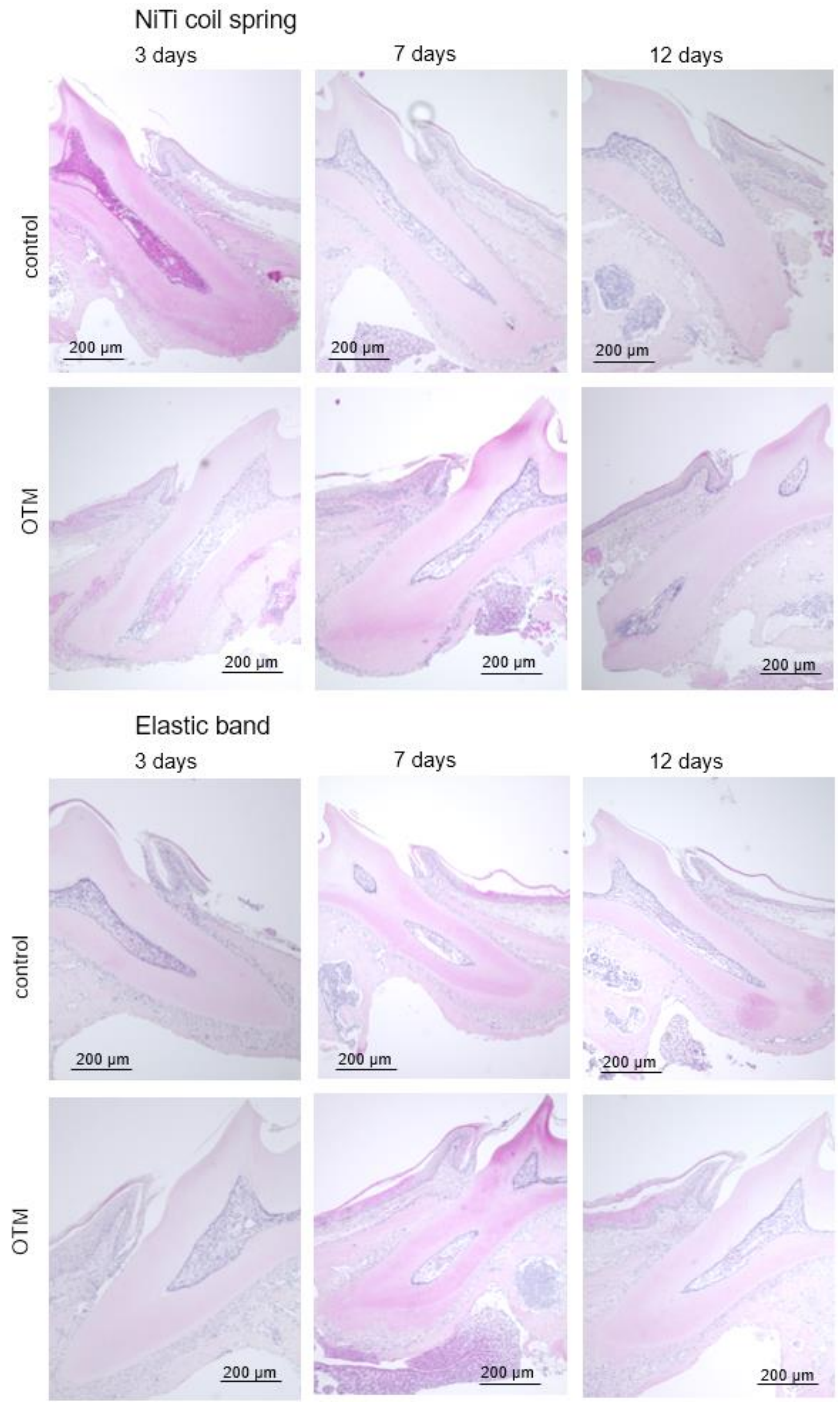

Supplemental Figure 4. Representative pictures of HE stainings of the first upper molar for determination of root resorptions. OTM = orthodontic tooth movement

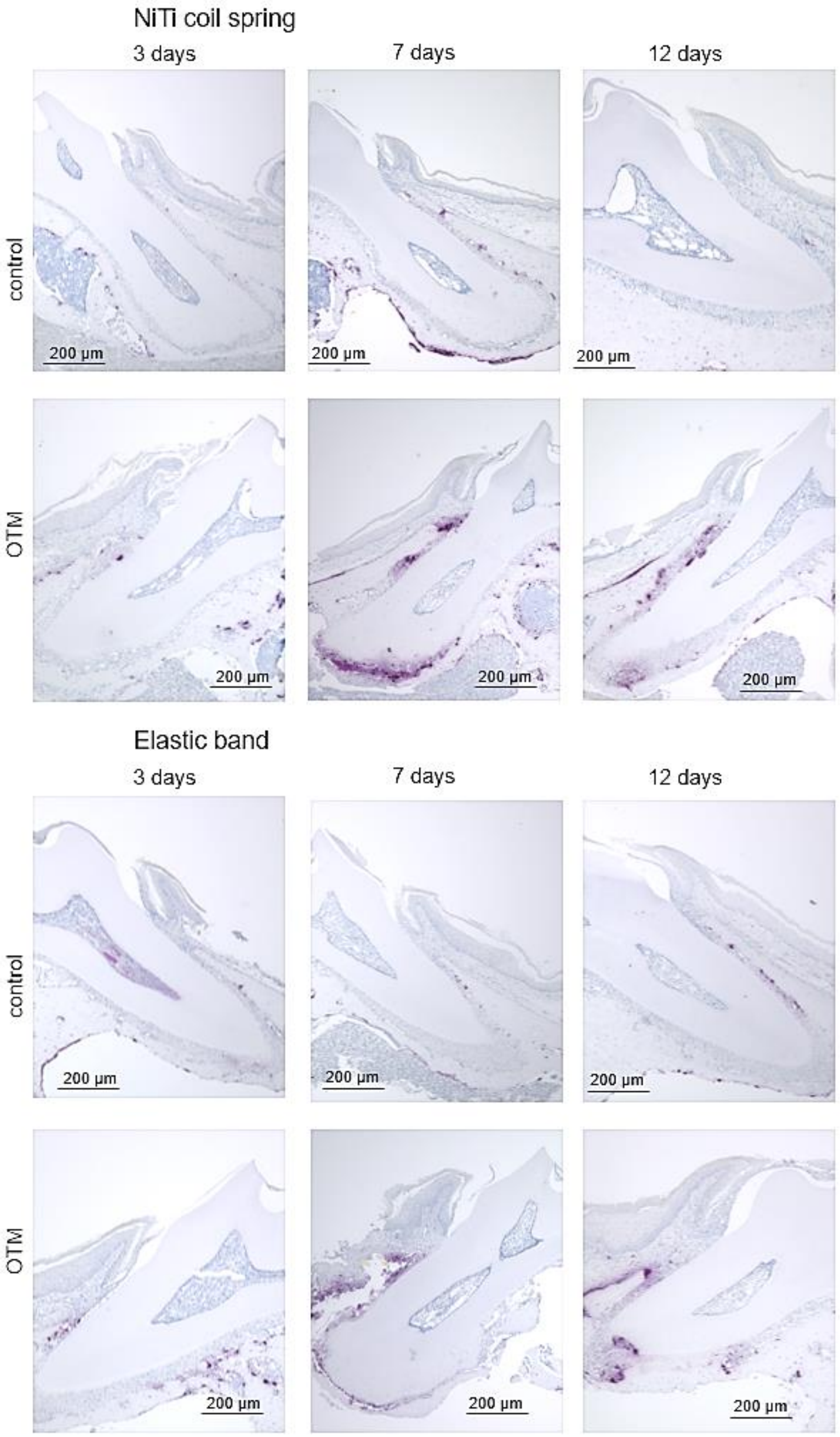

Supplemental Figure 5. Representative pictures of TRAP stainings of the first upper molar for determination of osteoclastogenesis. OTM = orthodontic tooth movement
